# Protein enrichment of the red macroalga *Palmaria palmata* using pulsed electric field and enzymatic processing

**DOI:** 10.1007/s10811-024-03338-3

**Published:** 2024-08-23

**Authors:** Ingrid Maribu, Marthe Jordbrekk Blikra, Karl-Erik Eilertsen, Kjetil Elvevold

**Affiliations:** 1 Department of Marine Biotechnology, Nofima – Norwegian Institute of Food, Fisheries and Aquaculture Research, Muninbakken 9-13, 9019 Tromsø, Norway; 2 Department of Processing Technology, Nofima – Norwegian Institute of Food, Fisheries and Aquaculture Research, Richard Johnsens gate 4, 4021 Stavanger, Norway; 3 Norwegian College of Fishery Science, Faculty of Biosciences, Fisheries and Economics, UiT – The Arctic University of Norway, 9037 Tromsø, Norway

**Keywords:** Pulsed electric field, Enzyme, Protein, *Palmaria palmata*, Processing, Macroalgae

## Abstract

The human population is steadily increasing and new alternative protein sources are necessary to secure food safety. There is a growing interest in macroalgae, or seaweed, as an alternative food source as they are rich in nutrients, minerals and carbohydrates. Among the diverse species of macroalga, *Palmaria palmata*, a red seaweed of growing interest due to its high protein content, represents a potential candidate for contributing to food security and animal feed. Novel methods are being investigated for extracting valuable components from seaweed, including protein. In this study, pulsed electric field (PEF) and enzymatic assisted extraction (EAE) were tested to investigate whether the methods, alone or in combination, were sufficient for protein extraction from *P. palmata*. The results show high extraction yields of dry matter consisting mostly of carbohydrates and ash when using EAE. The results point to a concentration of protein, including essential amino acids, in the pellet after extraction with a combination of PEF and EAE, or EAE alone. There is potential for the protein-rich pellet in animal feed. For the supernatant, there are potential uses within biostimulants.

**Supplementary Information:**

The online version contains supplementary material available at 10.1007/s10811-024-03338-3.

## Introduction

The human population is steadily increasing and is expected to exceed 9 billion by 2050. Therefore, from a food security perspective, it is important to find alternative food protein sources to meet the increasing demand (Béné et al. 2015). Today, plant protein makes up 65 %, whereas marine sources only comprise 6.5 % of the world’s supply of edible protein (Millward 1999; Béné et al. 2015). Macroalgae can be a good sources of nutrients, including minerals, carbohydrates, and proteins, with values depending on the species, location, and season of harvest (O’Connor et al. 2020). There is growing interest in macroalgae as an alternative protein source (Bjarnadóttir et al. 2018) and seaweed has a long tradition as food in Asian countries (Kadam et al. 2017). *Palmaria palmata* is a red seaweed with growing commercial interest due to its high protein content compared to other edible seaweed species (Stévant et al. 2023). It represents a potential candidate for supplementing animal feed and contributing to food security. However, the extraction of proteins from macroalgae is impeded by the robust cell wall structure, which hinders the bioavailability of these nutrients (Morais et al. 2020; O’Connor et al. 2020).

Seaweed proteins are mainly bound to the cell wall, reducing the protein’s bioaccessibility and digestibility for humans due to the lack of digestive enzymes (O’Connor et al. 2020). Therefore, extracting the proteins is considered to be an alternative to utilizing seaweed as a food source (Harnedy and FitzGerald 2013). Seaweeds have a complex and rigid cell wall comprising a range of macromolecules. The cell wall functions as a physical barrier for extracting proteins and other compounds and it is necessary to find processing strategies capable of breaking down this barrier (Deniaud et al. 2003). In *P. palmata*, the cell wall consists of mix-linked β-(1,3)/β-(1,4)-D-xylans and a minor amount of β-(1,4)-D-xylans and cellulose (Deniaud et al. 2003). These polysaccharides have a strong ionic interaction with proteins and a high viscosity in aqueous solutions, making it hard to extract proteins (Joubert and Fleurence 2008). This interaction also reduces the accessibility and digestibility of the proteins (Galland-Irmouli et al. 1999). Several studies have shown that processing with different techniques, such as autoclaving and boiling, can increase the digestibility of proteins from seaweed (Avanza et al. 2013; Mæhre et al. 2016a). The digestibility of seaweed proteins will vary from species to species and has been shown to be higher for red seaweeds compared to green and brown seaweeds (Misurcova et al. 2010). For red seaweeds, the digestibility of proteins has been reported to vary from 2 % to 90 % in vitro (Mæhre et al. 2016a) and *P. palmata* has a relative digestibility of 56 % (Galland-Irmouli et al. 1999).

There are limited extraction technologies that disrupt the cell wall of macroalgae to make the protein and other nutrients accessible (Echave et al. 2021). However, several novel processing techniques, such as enzyme-assisted extraction (EAE), ultrasound-assisted extraction, pulsed electric field (PEF), microwave-assisted extraction, and high hydrostatic pressure, aim to break down the cell wall, which results in the release of intracellular molecules (Echave et al. 2021). PEF is a non-thermal, non-chemical, energy-sufficient method that uses short pulses of high-voltage electric fields that enhance the permeability of the cell wall due to electroporation in the structure (Barba et al. 2015; Golberg et al. 2016; Matos et al. 2021). When this change in the membrane occurs, small pores in the structure are formed, which allows for an outflow of cell wall components. As the cell wall of seaweeds is very rigid, despite sufficient energy inputs, the outer wall may be left unaffected while there are generated pores in the cell membrane. In this case, larger molecules like protein will be entrapped within the structure, and only smaller compounds are free to diffuse through the structure. PEF may, therefore, be used in combination with other methods, like EAE, to achieve better permeabilization of the rigid structure (Robin et al. 2018; Steinbruch et al. 2023). EAE is used for seaweed to disrupt the cell wall structure, resulting in the release of intracellular components. Enzymes do this by breaking down specific polymer bonds (Puri et al. 2012; Nadar et al. 2018; O'Brien et al. 2022).

The aim of this study was to investigate whether the use of PEF and a polysaccharide degrading enzyme (Depol 793), either alone or in combination, would be suitable strategies for protein extraction from the red seaweed *P. palmata*. The methods and equipment described in this study are chosen based on their scalability.

## Materials and methods

### Raw material

*Palmaria palmata* was harvested in March 2023 in Kårvik, Norway (N69°52’05.8, E18°55’34.6). The plants were a mixture of tetrasporophytes and gametophytes of different sizes (approx. 20 – 30 thalli). The fresh *P. palmata* was shipped in seawater overnight on ice from Kårvik to Randaberg to keep it fresh and alive until processing. The seaweed was stored in tanks at NORCE, Randaberg, Norway, with constant flow through of fresh seawater at 8.4 ± 0.2 L min^-1^. The seawater in the tank was kept at 3.0 ± 0.1 °C to mimic the temperature of the seawater in Kårvik at the harvesting time. Before processing, the seaweed was rinsed with tap water to remove biofouling and epiphytes were manually removed. From each sample set, three batches of 10 g (w/w) *P. palmata* in 100 mL tap water (± 20 °C) (1:11) were used per treatment (henceforth referred to as 3 biological replicates).

### Pulsed electrical field treatment

The PEF treatment was conducted using a PEF Pilot Dual (Elea GmbH, Quakenbrück, Germany). equipped with an 8 dL batch chamber (electrode distance 8 cm). For the treatment, two different electrode voltages of 4 kV and 24 kV (effective field strength of 0.5 kV cm^-1^ (134.6 ± 50.2 J g^-1^) and 3 kV cm^-1^ (46565.5 ± 8618.0 J g^-1^), respectively) were applied to the seaweed batches (n = 3). The frequency, pulse width, and pulse count were kept constant at 30 Hz, 6 µs, and 800 counts, respectively, at room temperature (± 20 °C). After treatment with PEF, the seaweed batches were homogenized (both seaweed and water) using an IKA T25 digital Ultra Turaxx (IKA, China) at 3600 ± 200 rpm until a sufficient degree of mincing was achieved upon visual inspection, i.e., for 5 ± 2 min.

### Enzyme assisted extraction

**Fig. 1:**
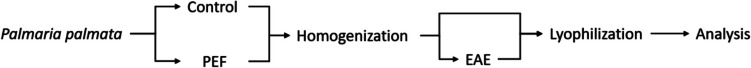
Experimental workflow

Prior to these experiments, it was conducted an enzyme screening to determine what enzyme to use for the EAE (Table S1). The selected enzyme, Depol 793 (Biocatalysts, United Kingdom; Batch number 27060), a mixture of β-glucanase, pectin lyase, and cellulase, was added to the homogenized samples constituting 1 % of the total volume. The enzymatic treatment was conducted at 40 °C for 1 h under constant stirring at 50 rpm (New Brunswick Incubator Shaker Innova 40, Fisher Scientific, USA). The samples were centrifuged at 10 000 × *g* for 20 min (Multifuge X3 FR, Thermo Fisher Scientific, USA), the supernatant and pellet were separated and both fractions were finally lyophilized (Gamma 2-16 LSCplus, Christ, Germany). The experimental workflow can be seen in Fig. 1.

### Dry matter and ash

The dry matter (DM) and ash content in the supernatant and the ash content in the pellets were determined through thermogravimetric analysis (TGA) using a TGA/DSC 3+ (Mettler Toledo, USA). The sample mass (approximately 30 mg wet supernatant and 4 mg lyophilized pellet) was first heated from 30 to 105 °C with a heating rate of 40 K min^-1^ and held at 105 °C for 10 min to determine the moisture content. The temperature was then increased to 600 °C with a heating rate of 10 K min^-1^ and held at 600 °C for 10 min to determine the ash content.

### Protein

Two copper catalyst tablets (Kjeltabs Cu/ 3.5, Nerliens Meszansky, Oslo, Norway) were added to approximately 0.01 g of lyophilized supernatant and pellet, which was hydrolyzed with 15 mL concentrated H_2_SO_4_ (Merck, Germany) in a heat block (Kjeltec system 2020 digestor, Tecator Inc, USA) at 420 °C for 1 h. The samples were then cooled and 30 mL of distilled water (ELGA Purelab Chorus 2^+^, Veolia Water, UK) was added to each sample before they were neutralized and titrated, and total nitrogen was measured using a Kjeltec 8400 (FOSS Analytics, Denmark). The amount of total protein was determined using the suggested conversion factor of 4.59 for red seaweeds (Lourenço et al. 2002). Individual nitrogen-to-protein conversion factors were calculated for the raw material and supernatant samples using Formula 1, derived from Bjarnadóttir et al. (2018).

1 $$Nitrogen-to-protein\;conversion\;factor=\frac{Sum\;Amino\;Acids\;\left(\%\right)}{Total\;Nitrogen\;\left(\%\right)}$$

### Amino acids

Approximately 40 mg of lyophilized supernatant was dissolved in 0.7 mL distilled water and 0.5 mL 20 mM DL-norleucine (Sigma Aldrich, USA). After the addition of 1.2 mL concentrated HCl (37 %) (Sigma Aldrich, USA), the samples were flushed with nitrogen for 10 s and placed in a heating cabinet at 105 °C for 26 h. Following hydrolysis, the samples were cooled, centrifuged at 18 000 × *g* for 5 min and 0.1 mL of each sample was evaporated under N_2_-gas until dry. The samples were dissolved in 1 mL lithium buffer (pH 2.2) and analyzed using a Biochrom 30+ amino acid analyzer (Biochrom Co., UK) with a lithium citrate equilibrated ion exchange column and post-column derivatization with ninhydrine. The results were analyzed using Chromeleon software (Dionex, USA). The A6407 and A6282 (Sigma Aldrich) amino acid standards were used for the identification and quantification of the amino acids.

### Sugars

A modified version of the method suggested by Englyst et al. (1994) was followed. Approximately 50 mg of lyophilized sample was soaked in 2.5 mL 12 M H_2_SO_4_, vortexed, placed in a water bath for 30 min at 35 °C, with vortexing at 5, 10 and 20 min. After adding 12.5 mL of distilled water, the samples were shaken and hydrolyzed in a boiling water bath for 1 h. One mL of hydrolyzate was mixed with 0.5 mL of the internal standard Allose (1 mg mL^-1^, Thermo Fisher Scientific), cooled on ice, and then mixed with 0.4 mL 12 M NH_3_ (VWR chemicals, USA). After adding 0.1 mL NH_3_NaBH_4_, samples were incubated at 40 °C for 30 min and mixed with 0.2 mL acetic acid (Merck). Then 0.5 mL methylimidazole (C_4_H_6_N_2_) (Sigma Aldrich) and 0.5 mL sample were mixed with 5 mL acetic anhydride, incubated for 10 min, followed by mixing with 0.9 mL absolute EtOH (Antibac AS, Norway), further incubation for 5 min and a final 5 min incubation after mixing with 10 mL distilled water. After mixing with 0.5 mL Bromophenol Blue solution (100 mg in 250 mL), samples were cooled in ice water, mixed with 5 mL 7.5 KOH, and incubated for 2 min. After mixing with 5 mL KOH (Sigma Aldrich) samples were placed at 4 °C until two phases had separated. The upper phase was added to GC vials. Standards including fucose, galactose, glucose, arabinose, rhamnose, mannose (all from Sigma Aldrich), and xylose (Merck), were diluted in 50 % benzoic acid (Merck). The samples were analyzed on an Agilent 8860 GC System with OpenLab Software (both from Agilent, USA)

### Statistical analysis

All results are presented as mean with the standard deviation of the biological replicates (*n* = 3, with technical replication (N = 3) of each biological replicate, unless otherwise stated. The error bars represent the combined standard deviation of both biological and technical replicates. One-way ANOVA was performed to test for significant differences between the sample groups using Minitab version 21.4.1. with a 95 % confidence interval. A Tukey post hoc test with a significance level of P < 0.05 was applied to test for variances when more than two sample groups were present.

## Results

### Dry matter and ash

After processing and centrifugation, the samples were divided into two fractions, a liquid fraction, hence referred to as “Supernatant”, and a solid fraction, hence referred to as “Pellet”. The DM content in the two fractions was greatly affected by processing and Table 1 summarizes the DM content in each fraction obtained from 10 g (w/w) *P. palmata*. The DM content in the raw material was 11.8 %. There was a significantly higher content of DM in the supernatant after PEF processing at both low (PEF1) and high voltage (PEF2), where 34 – 41 % of the total DM content was extracted into the supernatant, compared to the untreated control with a DM content of 23 %. When the samples were treated with enzyme, the DM content in the supernatant significantly increased to 73 %. There was no significant difference in DM content when the samples were treated with a combination of PEF and enzyme, compared to the sample only treated with enzyme.

**Table 1:** Extraction yields of dry matter content in the different fractions as mean percent ± standard deviation and the ash content as percent dry weight ± standard deviation (n = 3, with technical replication (N = 3) of each biological replicate) obtained from the starting material of 10 g (w/w) P. palmata. The samples: Control: soaking; Enzyme: treated with enzyme; PEF1 and PEF2: treated with low and high voltage; PEF1+E and PEF2+E: treated with low or high voltage followed by enzyme treatment

	Dry matter distribution (% of total DM)		Ash (% of fraction DM)	
	Supernatant	Pellet	Supernatant	Pellet
Control	23.3±3.3^C^	76.8±3.3^A^	42.9±4.7^B^	23.7±1.0^A^
Enzyme	73.5±1.8^A^	26.5±1.8^C^	38.4±1.5^BC^	22.0±0.6^B^
PEF1	34.7±4.1^B^	65.3±4.1^B^	43.9±5.8^B^	25.1±1.3^A^
PEF1+E	74.1±1.3^A^	25.9±1.3^C^	33.6±9.4^C^	20.3±1.2^B^
PEF2	41.3±6.9^B^	58.7±6.9^B^	53.7±5.8^A^	23.9±1.3^A^
PEF2+E	69.7±2.8^A^	30.3±2.8^C^	39.8±1.3^BC^	21.7±0.9^B^

Capital letters indicate significant statistical differences within groups

The DM samples contained a high amount of ash. Table 1 summarizes the ash content in the fractions as percent DM. The ash content in the raw material was as high as 22.9 % of the DM. The percentage of ash in the pellet was higher after PEF processing alone (~25 %) than after PEF and enzyme combined (~20 %). As the ash content increased in the supernatant, it decreased in the respective pellets.

### Protein and amino acids

**Fig. 2:**
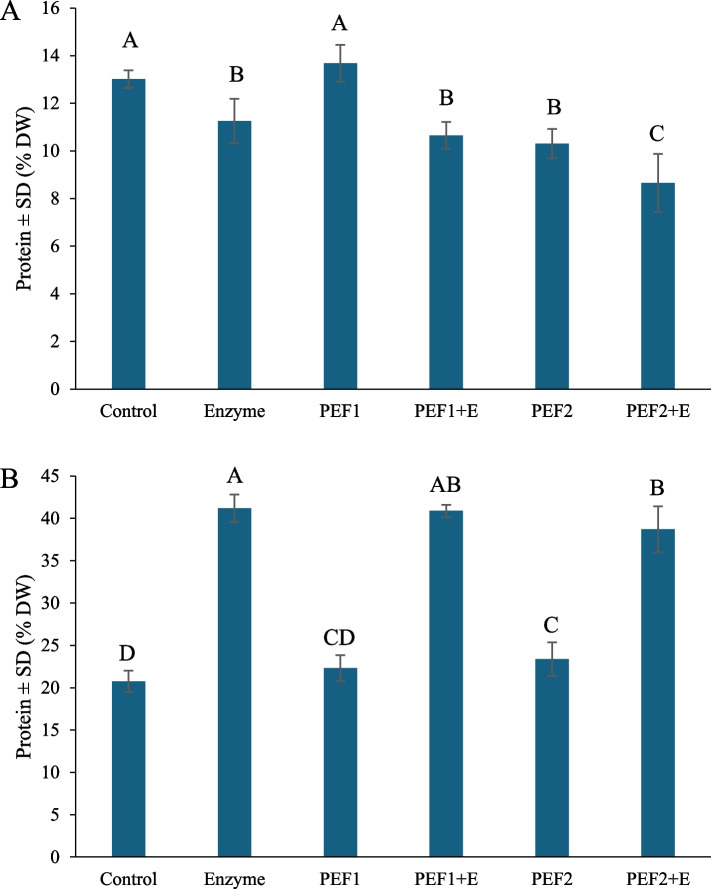
Protein content presented as percent dry weight ± standard deviation (n = 3, with technical replication (N = 3) of each biological replicate) in the supernatant (**A**) and pellet (**B**) after PEF and enzyme treatment. Capital letters indicate significant differences within groups. The samples: Control: soaking; Enzyme: treated with enzyme; PEF1 and PEF2: treated with low and high voltage; PEF1+E and PEF2+E: treated with low or high voltage followed by enzyme treatment

The percentage of protein in the DM in the supernatant and pellet is shown in Fig. 2A and B, respectively. The protein content in the supernatant from the control and the lower voltage PEF1 treatment sample was significantly higher than the other treatments (~13 %). The supernatant from the PEF2+E treatment had the lowest protein content (8.6 %). However, for the pellets, the samples treated with enzyme, and a combination of PEF and enzyme, PEF1+E and PEF2+E, had the highest protein content on a dry weight (DW) basis. This indicates a higher protein concentration in these pellets (38 – 41 %) compared to the control (20 %). The protein content in the pellet after the PEF2 treatment is statistically higher than the control sample, whereas the PEF1 treatment is not.

The amino acid (AA) composition of the raw material, as well as the supernatant of control, enzyme- and PEF-treated samples, is shown in Table 2 as mg AA g DW^-1^. The proportion of AA extracted into the supernatant ranged from 18 to 42 %, where PEF2 had the lowest extraction yield and PEF1+E had the highest. Of the extracted AA, 19 – 34 % were essential amino acids (EAA), which indicates that a substantial amount of EAAs remained in the pellet. Glutamic acid and aspartic acid were not extracted into the supernatant, except for the PEF 1 treatment, where a significantly higher amount of glutamic acid was extracted into the supernatant (32 %), which was higher than the control (29 %) and close to what was found in the raw material (38 %). Cysteine was efficiently extracted into the supernatant with various treatments, including soaking (control). The individual nitrogen-to-protein conversion factor for all fractions was calculated using Formula 1 and varied between 3.93 and 5.52 (Table 2).

**Table 2:** Amino acid (AA) composition in the supernatant from the control, enzyme and PEF treated P. *palmata*, as well as in the raw material. The data are given as mean mg AA g^-1^ DW ± standard deviation (n = 1, with technical replication (N = 3) of one biological replicate). The samples: Control: soaking; Enzyme: treated with enzyme; PEF1 and PEF2: treated with low and high voltage; PEF1+E and PEF2+E: treated with low or high voltage followed by enzyme treatment

	Raw material	Control	Enzyme	PEF1	PEF1+E	PEF2	PEF2+E
Histidine	3.44±0.60^A^	0.66±0.57^BC^	1.33±0.18^B^	0.54±0.47^BC^	1.50±0.46^B^	0.00^C^	0.58±0.51^BC^
Isoleucine	9.62±1.87^A^	2.97±0.20^BC^	4.08±0.65^BC^	3.17±0.38^BC^	4.33±0.65^B^	1.71±1.06^C^	2.79±0.32^BC^
Leucine	17.28±4.04^A^	5.16±0.50^B^	6.70±1.09^B^	5.64±0.74^B^	7.13±1.28^B^	2.98±1.84^B^	4.54±0.77^B^
Phenylalanine	10.51±2.35^A^	3.20±0.38^B^	4.18±0.75^B^	3.22±0.48^B^	4.49±0.75^B^	1.97±1.13^B^	2,88±0.12^B^
Methionine	5.39±1.43^A^	0.76±0.68^BC^	1.87±0.15^B^	0.15±0.26^BC^	1.88±0.33^B^	0.00^C^	0.63±0.55^BC^
Lysine	16.22±3.06^A^	4.52±0.59^B^	3.74±0.33^B^	4.63±0.36^B^	4.11±1.33^B^	3.04±0.36^B^	3.00±0.71^B^
Threonine	12.9±2.55^A^	5.52±0.56^BC^	8.02±1.32^BC^	6.27±0.48^BC^	8.46±1.11^B^	4.20±2.23^C^	6.39±0.72^BC^
Tryptophan	n. d	n. d	n. d	n. d	n. d	n. d	n. d
Valine	14.45±1.87^A^	4.11±0.64^BC^	5.87±1.17^BC^	4.64±0.31^BC^	6.34±0.96^B^	2.77±1.40^C^	4.12±0.71^BC^
Alanine	20.99±4.53^A^	13.00±1.84^B^	9.08±0.97^B^	10.51±0.89^B^	9.25±1.35^B^	11.24±2.82^B^	7.01±1.02^B^
Arginine	22.2±7.02^A^	6.20±1.24^B^	4.87±1.16^B^	5.31±0.97^B^	5.07±0.76^B^	4.15±1.60^B^	4.17±1.34^B^
Asparagine	n. d	n. d	n. d	n. d	n. d	n. d	n. d
Aspartic acid	20.37±4.10^A^	8.51±0.61^B^	10.33±1.62^B^	10.50±0.47^B^	11.27±1.60^B^	6.40±2.01^B^	8.30±1.11^B^
Cysteine	2.54±0.07^A^	3.58±0.49^A^	2.34±0.38^A^	3.39±0.55^A^	2.57±1.31^A^	3.58±0.68^A^	2.41±0.74^A^
Glutamine	n. d	n. d	n. d	n. d	n. d	n. d	n. d
Glutamic acid	38.51±9.50^A^	29.52±3.07^ABC^	19.52±0.72^BC^	32.03±0.29^AB^	20.40±4.65^BC^	25.21±5.17^BC^	17.51±2.69^C^
Glycine	17.46±3.31^A^	7.73±0.73^B^	8.22±0.96^B^	7.84±0.20^B^	8.43±1.33^B^	6.81±0.88^B^	6.68±0.76^B^
Proline	26.69±5.66^AB^	38.70±5.35^A^	18.51±0.97^B^	39.90±3.64^A^	19.07±5.85^B^	35.49±9.02^A^	19.53±3.84^B^
Serine	13.53±2.87^A^	4.63±0.65^BC^	7.59±1.53^BC^	4.67±0.21^BC^	8.19±1.06^B^	3.43±2.30^C^	5.83±0.51^BC^
Tyrosine	6.80±1.59^A^	0.99±0.88^B^	3.83±0.74^AB^	1.08±1.13^B^	4.20±0.88^AB^	1.15±1.98^B^	3.00±0.10^B^
Total amino acids	221.10±48.01^A^	121.40±13.53^B^	107.82±0.51^B^	118.33±0.07^B^	95.89±9.83^B^	96.49±6.01^B^	78.88±9.80^B^
% Protein	22.11±4.81^A^	12.14±1.35^B^	10.78±0.05^B^	11.83±0.01^B^	9.59±1.00^B^	9.65±0.60^B^	7.88±1.00^B^
% Kjeldahl Protein (4.59)	18.39±0.29^A^	13.02±0.37^B^	11.26±0.94^C^	13.68±0.78^B^	10.66±0.57^C^	10.31±0.62^C^	8.66±1.22^D^
Individual nitrogen-to-protein conversion factor	5.52	4.28	4.19	3.97	3.93	4.30	3.93

n.d = not detected

Capital letters indicate statistically significant differences within groups

### Sugars

The sugar content in the supernatants varied significantly following the different processing treatments, as shown in Table 3. The enzyme-treated samples, as well as those subjected to a combination of PEF and enzyme treatment (PEF1+E and PEF2+E), contained the highest levels of sugar, with the PEF2+E treatment resulting in sugars comprising 42 % of the DW. Galactose comprised between 2.7 % and 7.5 % of the DW across the samples. All samples treated with enzyme, alone or in combination with PEF, had relatively low galactose content (2.7 – 3.5 %). In contrast, glucose levels were significantly higher in the enzyme treated samples than in the control or samples treated with PEF alone. The highest xylose contents were found in the samples treated with either the combination of PEF and enzyme or with enzyme alone, with the sample PEF2+E exhibiting the highest xylose content at 26 % of its DW. Notably, arabinose, fucose, mannose, and rhamnose were not detected after any treatments.

**Table 3:** Sugar content in the supernatant and raw material given as g monosaccharide (100 g)^-1^ sample ± standard deviation (n = 1, with technical replication (N = 2) of one biological replicate). The samples: Control: soaking; Enzyme: treated with enzyme; PEF1 and PEF2: treated with low and high voltage; PEF1+E and PEF2+E: treated with low or high voltage followed by enzyme treatment

	Raw material	Control	Enzyme	PEF1	PEF1+E	PEF2	PEF2+E
Arabinose	n. d	n. d	n. d	n. d	n. d	n. d	n. d
Fucose	n. d	n. d	n. d	n. d	n. d	n. d	n. d
Galactose	5.0±0.4^AB^	7.0±1.5^AB^	3.5±0.7^AB^	7.1±1.1^AB^	2.8±0.5^B^	7.5±3.5^A^	2.8±0.7^B^
Glucose	2.9±0.3^B^	1.7±0.1^B^	13.7±2.0^A^	1.2±0.8^B^	12.8±0.6^A^	0.9±0.7^B^	12.0±0.4^A^
Mannose	n. d	n. d	n. d	n. d	n. d	n. d	n. d
Rhamnose	n. d	n. d	n. d	n. d	n. d	n. d	n. d
Xylose	27.0±2.3^A^	9.4±1.6^CD^	16.5±1.6^BC^	5.3±0.9^D^	19.0±3.4^B^	4.2±2.1^D^	26.9±5.3^A^
Total	34.9±3.0^A^	18.1±1.1^B^	33.8±0.9^A^	13.5±2.7^B^	34.5±3.8^A^	12.5±5.4^B^	41.7±4.8^A^

n.d = not detected

Capital letters indicate statistically significant differences within each group

## Discussion

*Palmaria palmata*, as well as other seaweed species, has the potential to become important nutrient sources for animal feed and future food security (Krogdahl et al. 2021). As mentioned earlier, seaweeds have a complex cell wall which makes nutrients, such as protein, inaccessible (O’Connor et al. 2020). PEF and EAE are some of the methods that are being investigated to overcome some of these challenges (Echave et al. 2021) and are considered more efficient, environmentally friendly, and safer than, e.g., solvent-based extraction techniques (Puri et al. 2012; Nadar et al. 2018).

In this study PEF processing was investigated in terms of extraction from *P. palmata*. There were no significant differences in the DM extracted between PEF1 and PEF2 treatments compared to the control. However, PEF2 treatment significantly increased the relative ash content in the supernatant (53 %) compared to all the other treatments investigated. A study conducted on the green alga *Ulva rigida* showed PEF processing was an efficient method for deashing the algae, where 82.5 % of the ash was extracted (Robin et al. 2018).

PEF processing can be used in combination with EAE to increase the permeabilization of the cell wall and increasing extraction yields (Steinbruch et al. 2023). When the material was treated with a combination of PEF and enzyme, the DM content extracted was significantly higher than for PEF processing alone, indicating a significant contribution from the enzyme. The results show that this supernatant consists mainly of ash and sugar, leaving a protein-rich pellet. The enzyme used in this study targets carbohydrates, where the enzyme will not affect the protein structures. Carbohydrates comprise up to 74 % of the DW in *P. palmata*, where the main sugars are xylose, galactose, and glucose (Deniaud et al. 2006). The extracted xylose is obtained from the degradation of xylans (Bajpai 2014), which are the main carbohydrates in *P. palmata* (Stévant et al. 2023). EAE increased the extraction of xylose significantly, compared to PEF processing, where the combination PEF2+E had the highest yield.

Algal carbohydrates are known to form complexes with proteins, impacting their bioavailability (Schiener et al. 2017). The protein content measured in the supernatants after the various treatments is low, indicating that the protein is retained in the pellets. Despite having the highest DM content in the supernatants, the samples treated with enzyme and the combination of PEF and enzyme had the lowest protein content of approximately 10 % DW. However, as large amounts of sugar and ash are extracted into the supernatant with these treatments, the pellets have a protein content as high as 38 – 41 % DW, which is significantly higher than the control sample with 20 % DW. Similarly, Aasen et al. (2022) reported an increase of protein from 12 % to 28 % in the pellet of *P. palmata* using the enzyme xylanase.

The protein content in this study is analyzed using the Kjeldahl method. Traditionally, the general conversion factor of 6.25 is used. However, this factor is known to overestimate the protein content in seaweeds due to the high contents of non-protein nitrogen (Angell et al. 2016; Mæhre et al. 2016a). Therefore, a conversion factor of 4.59 was used, which is specifically suggested for red seaweeds (Lourenço et al. 2002). Due to large geographical and seasonal variations in seaweeds (Galland-Irmouli et al. 1999), a specific conversion factor for the raw material used in this study was calculated. This factor was calculated at 5.52, indicating that the protein content in the raw material is underestimated. A conversion factor of 4.7 has previously been reported for *P. palmata* harvested in May (Bjarnadóttir et al. 2018)*.* In addition, a specific conversion factor was calculated for each supernatant, which varied from 3.39 – 4.30, as seen in Table 2. Bjarnadóttir et al. (2018) have previously reported conversion factors ranging from 2.5 – 4.1 in *P. palmata* processed with the enzyme xylanase.

The supernatants and pellets have several potential uses. Seaweed has been used as a soil enhancer for centuries (Khan et al. 2009). Seaweed extracts (both solid and liquid) are a complex mixture of compounds, often including minerals, polysaccharides, and polyphenols (Calvo et al. 2014). Several studies show that seaweed extracts can result in enhanced growth, shoot growth, and yield of several agricultural crops, i.e., biostimulant effects (Kumar and Sahoo 2011; Calvo et al. 2014). In addition to improving crops, seaweed extracts can help improve soil conditions by increasing the availability of minerals, such as potassium and phosphorus (Eyras et al. 2008). The results from this study point to supernatants rich in minerals and sugars and pellets rich in protein and minerals, both of which have potential as biostimulants.

In addition, seaweeds have a long tradition as food and feed ingredients (Mouritsen et al. 2024). The protein-rich pellet has potential as both a food and feed ingredient. For nutritional purposes, the ingredients should have high digestibility and high nutritional value (Krogdahl et al. 2021). Previous studies conducted on *P. palmata* show that processing of the material can increase the bioavailability of compounds, including protein (Mæhre et al. 2016a; Bjarnadóttir et al. 2018; Aasen et al. 2022). Mæhre et al. (2016a) reported that heat treatment made the structures less rigid, increased the protein bioaccessibility of the material, and increased the release of AA during in vitro digestion. The protein bioaccessability of seaweeds processed by non-thermal techniques, such as PEF, has so far not been reported. Interestingly, there was a visible change in the samples after PEF treatment, where the material became considerably softer. Based on the increased tissue softness, combined with the high extraction yield of sugar, PEF might have a similar effect on the material as heat treatment, resulting in increased bioavailability of the protein. However, this needs to be confirmed by in vitro digestion experiments. Moreover, this study shows a low extraction yield of AA, where EAA comprises approximately 19 – 34 % of the extracted AA in the supernatant. This indicates that a high portion of EAA remained in the pellet, increasing its nutritional value. Previous research by Mæhre et al. (2016a) showed that boiling significantly increased the proportion of EAA, including methionine and histidine, in the pellet of *P. palmata*. Bjarnadóttir et al. (2018) also found an increase in EAA content in the solid-phase fraction after EAE of *P. palmata*. Non-essential AA like glutamic acid and aspartic acid, which contributes to the umami flavor of seaweeds (Milinovic et al. 2021), was found to constitute up to 27 % of the total AA content in *P. palmata* (Schiener et al. 2017). Most of these AA were extracted into the supernatant and this will, therefore, influence the taste profile of both the supernatant and the pellet. A washed-out flavor can be both beneficial and disadvantageous, depending on the envisaged use. There is potential for the protein-rich pellet obtained from *P. palmata* as an ingredient in animal feed if other protein-rich material is included to compensate for some limiting AA, including histidine, methionine, and cysteine (Krogdahl et al. 2021).

### Methodological considerations

The sample size used in the experiments is low (10 g (ww) *P. palmata*), which can affect the processing as the whole sample might not have reached the set criteria during processing, as well as individual differences within each specimen may become more prominent. For PEF processing, a chamber with 8 dL capacity was used, where the sample size covered the surface area of the chamber. During PEF 1 processing with 4 kV cm^-1^, the samples had low conductivity resulting in a lower energy input with a high standard deviation (134.5 ± 50.2 J g^-1^), which will influence the variation of the parallels. Further investigation with PEF for extraction should investigate the effect of several PEF settings and sample sizes.

## Conclusion

The results indicate that PEF processing is not suitable for protein extraction under the conditions tested in this study as very little protein was extracted into the supernatant. However, when PEF processing is combined with enzymatic treatment, the resulting supernatant is rich in sugar and minerals, while the pellet is enriched with protein. This presents a promising potential for the utilization of the supernatants and the protein-rich pellet as ingredient for animal feed. In addition, PEF processing will have an impact on the structural integrity of the components left in the pellet, which might make the proteins more bioavailable. However, this hypothesis needs to be validated in further studies. In addition to PEF, other processing methods, including ultrasound, could be explored for their efficacy in disrupting cell walls and facilitating the release of intracellular components. Other enzymes might also be interesting to look further into with the combination of PEF, such as xylanase, based on its efficiency in other studies on degradation of components in *P. palmata*. Future research should aim to optimize these combined processing techniques to maximize the extraction and bioavailability of valuable compounds from macroalgae.

## Supplementary Information

Below is the link to the electronic supplementary material.

Supplementary file1 (DOCX 21 KB)

## Data Availability

Data are available from the corresponding author upon reasonable request.
