# Supplementary material for: Protein enrichment of the red macroalga Palmaria palmata using pulsed electric field and enzymatic processing
Source: J Appl Phycol. 2024 Aug 23;36(6):3665–73. doi: 10.1007/s10811-024-03338-3 (PMC11659382; doi:10.1007/s10811-024-03338-3)
Supplement: Supplementary file 1 — Supplementary file1 (DOCX 21 KB) [file 10811_2024_3338_MOESM1_ESM.docx]

## Supplementary information - Protein enrichment of the red macroalgae *Palmaria palmata* using pulsed electric field and enzymatic processing

*Corresponding author: [Ingrid.maribu@nofima.no](mailto:Ingrid.maribu@nofima.no)

Prior to this study, an enzymatic screening was conducted on the material to determine what enzyme to use. This was determined based on the protein extraction efficiency determined by the Pierce Protein assay. The enzymes tested and the extracted protein content can be seen in Table S1.

Table S1. Enzymatic screening of different enzymes and their protein extraction efficiency from Palmaria palmata is given in µg/mL. The enzymatic activity, the producer of the enzyme, and the batch number for each enzyme are given. The control sample is a sample treated the exact same way without the addition of enzyme.

| Enzyme | Protein (µg/mL) | Activity | Producer | Batch number |
| --- | --- | --- | --- | --- |
| Bromelain | 175 | Non-specific cystein protease | Ultra bio-logics (Montreal, Canada) | BR1200-K10211 |
| Corolase 7089 | 100 | Neutral protease, bacterial endopeptidase (metallo- and serine- protease) | AB Enzymes (Darmstadt, Germany) | F181360ST |
| Depol 692 | 603 | Endo-galacturonase, cellulase | Biocatalysts (London, United Kingdom) | 21682 |
| Depol 793 | 513 | Beta-glucanase, pectin lyase, cellulase | Biocatalysts (London, United Kingdom) | 27060 |
| Endocut 01 | 109 | Broad specificity endo-protease | Tailorzyme (Herlev, Denmark) | 1043 |
| FoodPro PNL | 97 | Metallo neutral endopeptidase, | DANISCO (Copenhagen, Denmark | 2863924615 |
| Rohalase | 97 | Beta-1,3-glucanase | AB Enzymes (Darmstadt, Germany) | R206637ST |
| Viscozyme | 228 | Beta-glucanase, xylanase, cellulase, hemicellulase, arabanase | Novozymes (Copenhagen, Denmark) | Not available |
| Control | 96 |  |  |  |
